# Compressive Strength Prediction of Rubber Concrete Based on Artificial Neural Network Model with Hybrid Particle Swarm Optimization Algorithm

**DOI:** 10.3390/ma15113934

**Published:** 2022-05-31

**Authors:** Xiao-Yu Huang, Ke-Yang Wu, Shuai Wang, Tong Lu, Ying-Fa Lu, Wei-Chao Deng, Hou-Min Li

**Affiliations:** 1 School of Civil Engineering, Architecture, and Environment, Hubei University of Technology, Wuhan 430068, China; 102000669@hbut.edu.cn (X.-Y.H.); luf77@126.com (Y.-F.L.); dengweichao@petalmail.com (W.-C.D.); 2 Wuhan Construction Engineering Company Limited, Wuhan 430056, China; wukeyang0623@163.com (K.-Y.W.); wangshuai@wceg.com.cn (S.W.); lutong1@wceg.com.cn (T.L.)

**Keywords:** compressive strength, ANN, concrete, prediction, algorithm

## Abstract

Conventional neural networks tend to fall into local extremum on large datasets, while the research on the strength of rubber concrete using intelligent algorithms to optimize artificial neural networks is limited. Therefore, to improve the prediction accuracy of rubber concrete strength, an artificial neural network model with hybrid algorithm optimization was developed in this study. The main strategy is to mix the simulated annealing (SA) algorithm with the particle swarm optimization (PSO) algorithm, using the SA algorithm to compensate for the weak global search capability of the PSO algorithm at a later stage while changing the inertia factor of the PSO algorithm to an adaptive state. For this purpose, data were first collected from the published literature to create a database. Next, ANN and PSO-ANN models are also built for comparison while four evaluation metrics, MSE, RMSE, MAE, and $R^{2}$, were used to assess the model performance. Finally, compared with empirical formulations and other neural network models, the result shows that the proposed optimized artificial neural network model successfully improves the accuracy of predicting the strength of rubber concrete. This provides a new option for predicting the strength of rubber concrete.

## 1. Introduction

Concrete is one of the most widely used building materials in the construction industry [1]. The global construction industry is challenged by the high cost and environmental pollution caused by concrete; meanwhile, there are increasing demands on the strength and durability of concrete. Thus, the traditional concrete is being replaced by concrete with better performance. Meanwhile, the rubber tires have vast output from other waste materials due to their excellent strength, low cost, and easy availability. In addition, adding rubber scraps to the cementitious composites can reduce carbon dioxide emissions which is environmentally friendly [2,3]. Therefore, the addition of rubber to concrete (rubberized concrete) becomes a feasible plan [4,5,6,7,8,9,10]. However, the compressive strength of rubberized concrete is lower than that of plain concrete [11]. This decrease is caused by variables including the ratio of aggregate (reduced 85% and 65% of compressive strength, when the coarse aggregate and fine aggregate are fully replaced by rubber, respectively) [8,11,12], size, and shape of rubber [12,13]. 

Machine learning, which can learn from given data and make accurate predictions through complex systems, includes the support vector machine model (SVM), random forest model (RF), fuzzy logic (FL), artificial neural network (ANN) model, etc. [14]. Machine learning techniques have been used extensively in the study of concrete, for example, Nasrollahzadeh. K and Nouhi. E [15] developed a fuzzy inference system (FIS) model that was used to investigate the compressive strength and strain capacity of axially loaded fiber-reinforced polymer concrete columns, and it was successfully demonstrated to be superior to existing models. Mahmood Ahmad et al. [16]. used three machine learning models, AdaBoost, RF, and decision tree (DT), to predict the compressive strength of concrete at high temperatures and demonstrated the applicability of supervised learning methods to study the compressive strength of concrete at high temperatures. Artificial neural network models are the most widely used of the many machine learning models due to their powerful nonlinear ability to learn from numerous complex situations. Current research on the strength of rubber concrete using artificial neural networks is proving successful, for example, Abdollahzadeh et al. [17] used 20 data to build a neural network model to predict the strength of rubber concrete, with the structure of the network set to 3–1–1, and the final accuracy of the model was calculated to be 96%. El-Khoja A. et al. [9] built a neural network model using 287 data with input variables set to five. The accuracy of the model was calculated to be 91%, while the optimal structure was determined to be 5–10–1. Nyarko et al. [7] used rubber concrete data to build a deep neural network model with the structure 9–3–2, and demonstrated that deep neural networks could be an alternative option for predicting the strength of rubber concrete. From the literature, it is known that there are fewer studies using intelligent algorithms for optimization. Intelligent algorithms achieve optimal results when solving complex nonlinear problems. Conventional ANN models tend to fall into local extremum with large datasets, while the strength of rubber concrete is influenced by a variety of nonlinear factors, so the use of intelligent algorithms is appropriate. Particle swarm optimization (PSO) algorithms were widely used in model optimization due to their fast convergence of iterations and simplicity of operation [18,19,20]. However, due to the fixed value of the inertia factor of the PSO algorithm, this makes the search space of the particles small and causes the algorithm to easily fall into local extremes late in the iteration. The simulated annealing (SA) algorithm was a global search algorithm with a good ability to search globally and accept poorer values, so it could jump out of local extremum to obtain the global best value [21]. Hybrid algorithms have not been used in current research on the strength of rubber concrete using neural networks. Therefore, an adaptive simulated annealing particle swarm optimization (ASAPSO) algorithm is developed in this study. 

In summary, this study developed an ANN model based on ASAPSO algorithm optimized to predict the strength of rubber concrete. This model could achieve higher accuracy than conventional ANN model and ANN model optimized by a single algorithm under a large dataset. Thus, a database was first created and analyzed to filter out 11 variables that sensitivity factor analysis was performed on. Then, three different models, ANN models, ANN models optimized by PSO (PSO-ANN) algorithm, and the ANN models optimized by ASAPSO (ASAPSO-ANN) algorithm, were built. The database data were fed into the three models for training and testing. Finally, the performances of the three models were evaluated statistically, and their computational results were compared and analyzed.

## 2. Database Description and Analysis of Variables

A reliable database is essential for the successful training of machine learning models. Without reliable data, the training results do not reflect the actual situation and eventually lead to model training failure. This study, therefore, proposes the following treatments in the process of building a database of rubber concrete:

Firstly, an adequate database does not only require a large amount of data, but also a comprehensive range of input and output variables. Therefore, data need to be collected from previously published literature to build the database.

Secondly, concrete is a composite material. As the material has a significant impact on strength, different materials need to be distinguished during data collection to make the network more capable of learning. For example, the compressive strength values and mechanisms of action of concrete mixtures formed with ordinary silicate cement are different from those of concrete mixtures formed with cement replacement materials. It is also necessary to divide the dimensions of the rubber material; this is because the rubber material replaces different objects.

Thirdly, the size and shape of the concrete may vary. Therefore, the specimen size and shape are considered in database creation. There is a need to standardize the units in which the data are collected. Therefore, except for the specimen dimensions in mm and the compressive strength in MP, the units for all data in this study are kg/m^3^. In addition, the rubber concrete samples in this study were all on a 28-day curing cycle.

**Figure 1:**
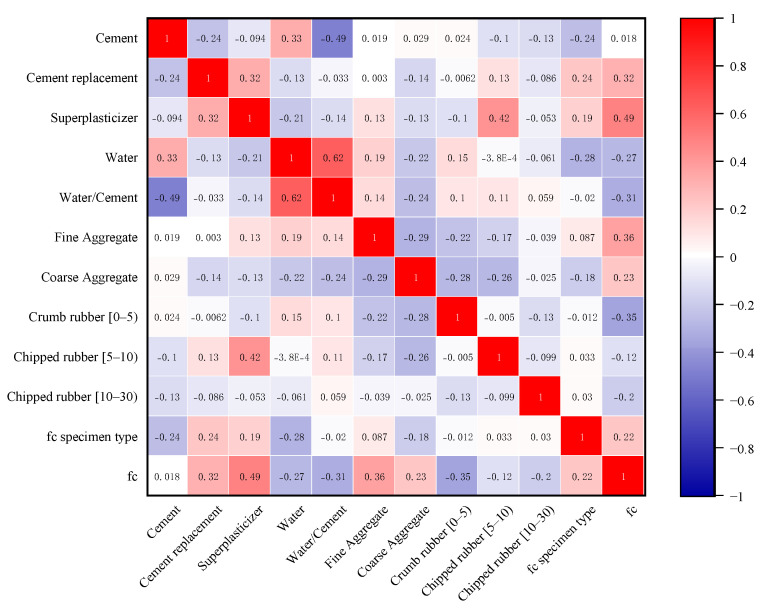
Heat map of the correlation for each variable.

In summary, a database of 307 sets of experimental data has been developed in this paper, where the data were derived from the published literature [6,8,10,22,23,24,25,26,27,28,29,30,31,32,33,34,35,36,37,38,39,40,41,42,43,44,45,46,47,48,49,50,51,52]. The database was divided into two parts, of which 277 groups were used for training and the remaining 30 groups were used for testing. Eleven input variables were used in this paper; these include cement content, water content, water–cement ratio, cement replacement material content, coarse aggregate content, fine aggregate content, crumb rubber content, flake rubber content, admixture addition, and specimen shape. The output variable was the compressive strength of the rubber concrete at 28 days. The statistical analysis of the input and output variables of the database is shown in Table 1. It is important to note that the interdependency of the model is an important parameter as it can lead to poor performance of the model [53]. The various parameters used as inputs can be interdependent, and such problems are known as “multicollinearity problems” [54]. It has been suggested in the literature that to develop an accurate model, the correlation between the two input parameters should be less than 0.8 [55]. A heat map of the correlation coefficients of the input and output variables in this study is shown in Figure 1. Table 2 shows that the correlation coefficients between the parameters are all less than 0.8, thus reducing the effect of multiple collinearities.

## 3. Sensitivity Factor Analysis of Input Variables

**Figure 2:**
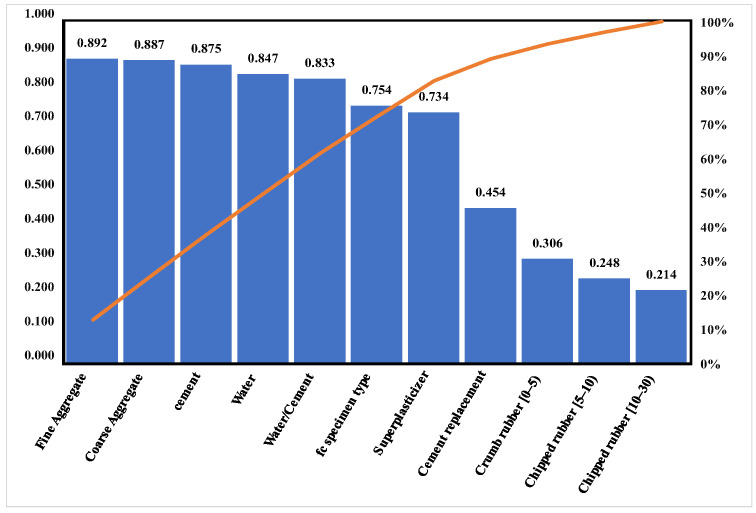
Analysis of sensitivity factors.

Sensitivity factor analysis (SA) is an effective method for measuring the influence of model input parameters on output parameters. The sensitivity factor analysis allows the input parameters to be filtered, thus reducing the complexity of the model, and saving time in model calculations. To achieve this, the cosine amplitude method (CAM) can be adopted [56]. The expression for this method is as follows:

(1) $$R_{ij}=\frac{\sum_{k=1}^{n}x_{ik}x_{jk}}{\sqrt{\sum_{k=1}^{n}x_{ik}^{2}\sum_{k=1}^{n}x_{jk}^{2}}}$$

where $x_{i}$ denotes the input parameter; $x_{j}$ denotes the output parameter; n indicates the number of data; $R_{ij}$ means the strength of the relationship. The values of $R_{ij}$ between the strength of the rubber concrete and the input parameters are shown in Figure 2. As can be seen from the graph, the most significant influence on the strength of the rubber concrete is the fine aggregate, followed by the coarse aggregate, while the smallest impact is on the largest particle size of the rubber sheet.

## 4. Methods

Artificial neural networks are used in various fields because of their powerful nonlinear capabilities. The simulated annealing (SA) algorithm is the same as the particle swarm optimization (PSO) algorithm—both are intelligent algorithms, but the SA algorithm is physically inspired and has better global search capabilities. The particle swarm algorithm is a swarm heuristic algorithm which is computationally simple and easy to operate but prone to local extremum. The ASAPSO algorithm and ANN model used are described in detail in this section.

### 4.1. Artificial Neural Network

**Figure 3:**
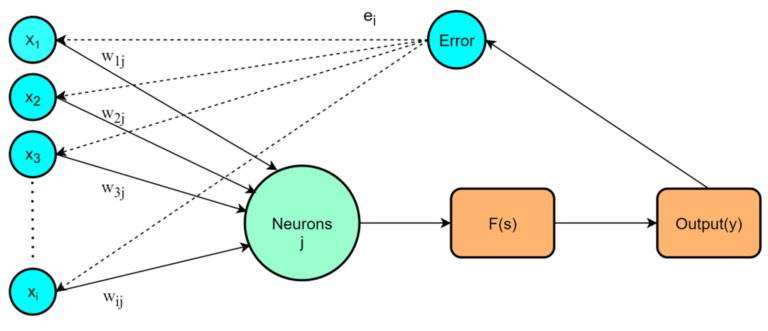
Backpropagation neural network structure.

The ANN is one of the most used machine learning techniques for predicting the compressive strength of concrete. ANN can be thought of as a data processing operation or as a black box that produces the appropriate output based on the input data [57]. A backpropagation network is the most commonly used network structure and it adjusts weights and biases by backpropagation of errors, eventually reducing the errors to an acceptable state [58]. A backpropagation neural network is composed of an input layer, a hidden layer, and an output layer. In addition to the input layer, the neurons of the hidden layer and output layer are composed of weights, biases, and activation functions. The structure of the backpropagation neural network is shown in Figure 3.

In Figure 3, $x_{i}$ represents input variables, $w_{ij}$ represents the connection weight. The expressions of s and neural network output y are shown in Equations (2) and (3) [59].

(2) $$d=\overset{n}{\underset{i=1}{\sum }}w_{ij}x_{i}+b$$

**Figure 4:**
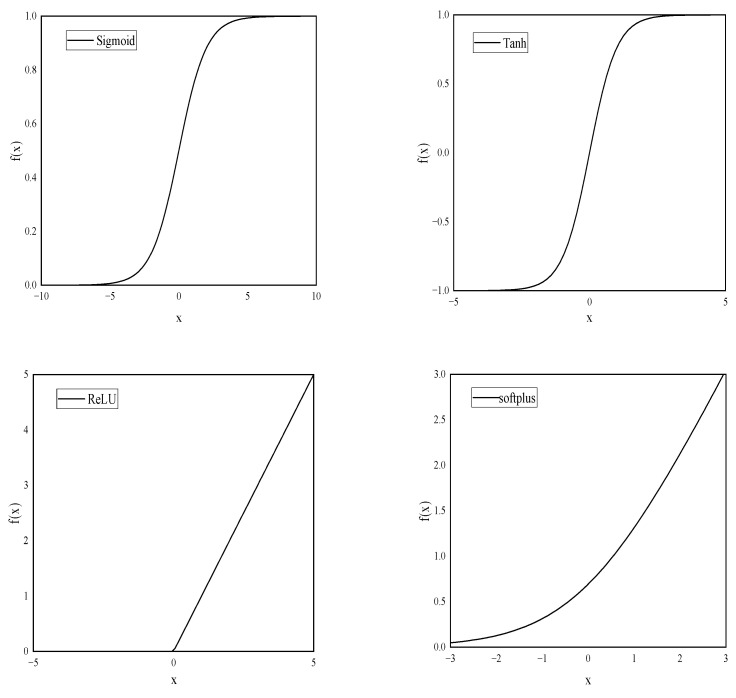
Four activation functions.

(3) Y=F(d)

where *b* is the bias, and *d* is the sum of weights and biases. The role of the bias is to increase or decrease the influence of the activation function. *F* denotes the activation function, which allows the neural network to approximate any nonlinear function and thus can be applied to a wide range of nonlinear models. There are various activation functions for neural networks, among which the commonly used transfer functions are sigmoid, softplus, tanh, and ReLU. An image of a commonly used activation function is shown in Figure 4.

Since the backpropagation network can constantly adjust the weights and biases based on the results, the weights and biases are updated by the following formula [59]:

(4) $$w\left(i+1\right)=w\left(i\right)-\alpha \frac{\partial E\left(i\right)}{\partial w\left(i\right)}$$

(5) $$b\left(i+1\right)=b\left(i\right)-\alpha \frac{\partial E\left(i\right)}{\partial b\left(i\right)}$$

where α indicates the learning rate; w(i) and b(i) denote the vector of connection weights and bias vectors between the layers of the ith  iteration, respectively; $\frac{\partial E\left(i\right)}{\partial w\left(i\right)}$ and $\frac{\partial E\left(i\right)}{\partial b\left(i\right)}$ denote the error gradient of the output error of the ith  iteration for each weight and bias, respectively; E(i) is the error in the output of the ith  iteration of the network, with the following functional expression [59]:

(6) $$E\left(k\right)=\sqrt{\frac{1}{n}\sum_{i=1}^{n}{\left|e_{i}\right|}^{2}}$$

where $e_{i}$ indicates the error between the actual value and the predicted value; n indicates the total amount of data.

The number of neurons contained in the hidden layer (*h*) is an important parameter of artificial neural networks. However, there is no specific calculation method for the number of neurons contained in the hidden layer. Usually, the approximate range is determined by an empirical formula, and then the optimal number of neurons is determined according to the trial-and-error method. The expression is given in Equation (7).

(7) $$h=\sqrt{m+n}+a$$

where *m* is the number of nodes in the input layer; *n* is the number of nodes in the output layer, a∈(1,10).

To reduce the prediction error and improve the training efficiency, it is necessary to normalize the training data and the verification data. The specific expression for the normalization function is as follows:

(8) $$y=\frac{\left(y_{max}-y_{min}\right)\left(x-x_{min}\right)}{x_{max}-x_{min}}+y_{min}$$

where *y* is the normalized value; $y_{max}$ and $y_{min}$ are the maximum and minimum values of the normalized range, respectively, usually taking the values [0,1]; *x* is the value of the input variable; $x_{max}$ and $x_{min}$ are the maximum and minimum values of the variables, respectively.

There are several classic algorithms in the training phase of ANN, namely, adaptive learning rate gradient descent algorithm, gradient descent algorithm, momentum gradient descent algorithm, conjugate gradient algorithm, and Levenberg–Marquardt (LM) algorithm [60]. The choice of the algorithm needs to depend on the situation. The LM algorithm is a classic backpropagation algorithm with fast convergence speed and high precision. The LM algorithm was chosen for this study.

### 4.2. Particle Swarm Optimization Algorithm

Similar to the genetic algorithm (GA), the PSO algorithm is also a new type of iterative algorithm based on the swarm evolution algorithm [61]. The PSO algorithm was proposed by Kennedy and Eberhar in 1995 [62]. Particles can be used to simulate individual birds, and the optimal path to find food is the search process of each particle, so each particle may be a candidate solution to a problem. Particles have only two properties: velocity and position, which are constantly updated through information interactions within the population. When any particle in the population finds the global optimum position, the other particles in the population update their velocity and position according to the optimum position and move closer to the optimum position [63]. The particle swarm optimization algorithm is widely used in various fields due to its simple operation and fast convergence [64]. The particle swarm algorithm first initializes a group of random particles. Assuming that the total number of particles is n, the position and velocity of the *i*th particle in d-dimensional space are expressed as follows:

(9) $$v_{i}^{d}\left(t\right)=\omega v_{i}^{d}\left(t-1\right)+c_{1}r_{1}\left(p_{best}^{d}-x_{i}^{d}\left(t\right)\right)+c_{2}r_{2}\left(G_{best}^{d}-x_{i}^{d}\left(t\right)\right)$$

(10) $$x_{i}^{d}\left(t\right)=x_{i}^{d}\left(t-1\right)+v_{i}^{d}\left(t\right)$$

where $v_{i}^{d}\left(t\right)$ is the speed of the particle at time *t*; $v_{i}^{d}\left(t-1\right)$ represents the velocity of the particle at time *t* − 1; $x_{i}^{d}\left(t\right)$ is the position of the particle at time *t*; $x_{i}^{d}\left(t-1\right)$ is the position of the particle at time *t* − 1; $p_{best}^{d}$ denotes the local optimum and $G_{best}^{d}$ denotes the global optimum; ω means inertia factor. The more significant the inertia factor, the better the global search, and the smaller the inertia factor, the better the local search [65]. $r_{1}$ and $r_{2}$ are random numbers between 0 and 1. $c_{1}$ is the individual learning factor, which represents the ability of the individual to search for the optimal solution, while $c_{2}$ is the social learning factor, which means the ability of the group to search for the optimal solution. Learning factors $c_{1}$ and $c_{2}$ are usually set as 2. If $c_{1}=0$, then the particle is considered to have only social learning ability; at this time, the particle has the ability of extended search and faster convergence speed but lacks the ability of local search and is prone to fall into the problem of local optimal solution on complex problems. If $c_{2}=0$, then the particle is considered to have the only cognitive ability; at this point, the particle behaves similar to a blind random search and converges slowly, which makes it less likely to eventually obtain the optimal solution.

### 4.3. Adaptive Particle Swarm Optimization

The inertia factor is an essential parameter in the PSO algorithm, which represents the effect of the velocity of the previous generation of particles on the speed of this generation of particles. For ordinary PSO algorithms, the inertia factor is static, but the static inertia factor is often not well adapted to the current environment and balances local search and global search. To improve the optimization ability of the PSO algorithm and reduce the probability of falling into the local extremum value, this study adopts a nonlinear decreasing method to optimize the inertia factor, and the specific expression is as follows [66]:

(11) $$\omega =\left(\omega_{max}-\omega_{min}-d_{1}\right)e^{\frac{1}{1+\frac{d_{2}*t}{T}}}$$

where $\omega_{max}$ and $\omega_{min}$ are the maximum and minimum values of the initial inertia factor, respectively. When $\omega_{max}$ = 0.95 and $\omega_{min}$ = 0.4, the algorithm’s performance will be significantly improved [67]. The control factors are $d_{1}$ and $d_{2}$, and the primary function is to control the inertia factor ω between $\omega_{max}$ and $\omega_{min}$; *t* is the current number of iterations, and *T* is the maximum number of iterations.

The improved algorithm allows the particles to have a larger inertia factor at the beginning of the iteration. This enhances the global search ability of the particle and reduces the probability of falling into a local optimum solution. As the number of iterations increases, the inertia factor gradually decreases. At the end of the iteration, the particle has a small inertia factor, which allows the particle to converge quickly to the global optimal solution, increasing the probability of obtaining the global optimal solution.

### 4.4. Adaptive Simulated Annealing Particle Swarm Optimization

The SA algorithm is a global optimization algorithm inspired by the metal annealing mechanism [56]. The SA algorithm consists of two processes, the Metropolis algorithm, and the annealing. The Metropolis algorithm is the basis for simulated annealing, where the objective function is allowed to degenerate over a range of values in the search for the optimum, allowing it to jump out of the local extremes and find the globally optimal solution. The primary process of the SA algorithm is as follows:

(1) Initialize the annealing temperature *T*, generate the initial solution $x_{0}$, and calculate the corresponding objective function value $F\left(x_{0}\right)$.
(2) *Set T* = *KT*, where *K* is the temperature drop rate, K∈(0,1).
(3) Apply random perturbation to the current solution $x_{0}$ to generate a new solution $x_{1}$ and calculate the corresponding objective function value $F\left(x_{1}\right)$, then the difference between the two objective functions is ∆*F = F(*$x_{1}$*) − F(* $x_{0}$*)*.
(4) If Δ*F* < 0, then accept the new solution as the current solution, otherwise, obtain the new solution as the current solution according to probability *exp* (*−*ΔF/KT).
(5) After the solution is obtained, whether the number of iterations is reached is judged. If the number of iterations is not reached, go back to steps 3 and 4. If it is reached, it is judged whether the termination condition (∆*F* < 0) is met. If the condition is met, output the result; otherwise, go back to step 2.

**Figure 5:**
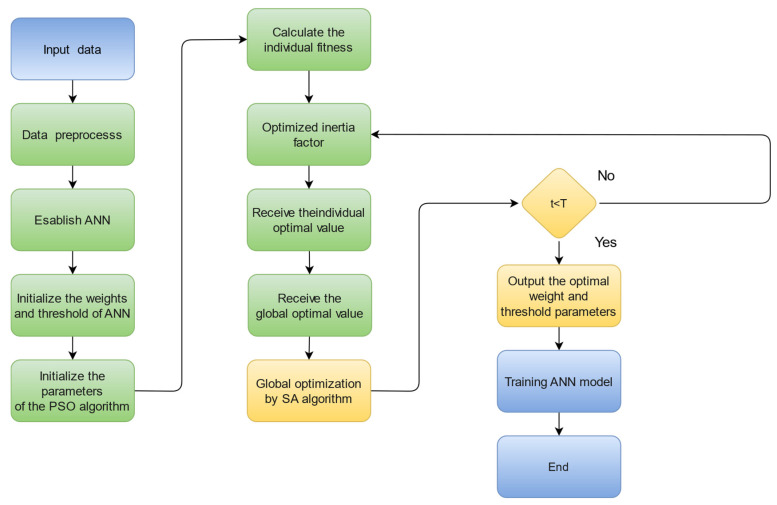
Workflow of the ASAPSO-ANN model.

The SA algorithm was introduced into the adaptive particle swarm optimization algorithm because the PSO algorithm is prone to local extremes late in the iteration, while the global search capability of the SA algorithm is stronger and can effectively compensate for the shortcomings of the PSO algorithm. The workflow of the ASAPSO-ANN model developed in this study is shown in Figure 5.

## 5. Evaluation of the Model

In the present work, four statistical criteria are used to evaluate the accuracy of neural network models, namely, mean square error (MSE), root mean square error (RMSE), mean absolute error (MAE), and coefficient of determination ($R^{2}$). The coefficient of determination ($R^{2}$) is widely used in regression problems [68]. Its main function is to evaluate the correlation between the actual target and the output target [69]. RMSE and MAE are used to evaluate the average error between the real target and the output target [70,71,72]. The accuracy of the model gradually improves as $R^{2}$ approaches 1, while MSE and MAE approach 0. The expressions of these four evaluation indicators are as follows [73,74]:

(12) $$R^{2}=\frac{\sum_{k=1}^{N}\left(q_{0,k}-\bar{q_{0}}\right)\left(q_{t,k}-\bar{q_{t}}\right)}{\sqrt{\sum_{k=1}^{N}{\left(q_{0,k}-\bar{q_{0}}\right)}^{2}\sum_{k=1}^{N}{\left(q_{t,k}-\bar{q_{t}}\right)}^{2}}}$$

(13) $$MAE=\frac{\sum_{k=1}^{N}\left|q_{0,k}-q_{t,k}\right|}{N}$$

(14) $$\;MSE=\frac{1}{N}\sum_{k=1}^{N}{\left(q_{0,k}-q_{t,k}\right)}^{2}$$

(15) $$RMSE=\sqrt{\frac{1}{N}\sum_{k=1}^{N}{\left(q_{0,k}-q_{t,k}\right)}^{2}}\;$$

where *N* is the number of samples; $q_{0}$ is the actual target value; $\bar{q_{0}}$ is the real target average value; $q_{t}$ represents the output target value; and $\bar{q_{t}}$ represents the output target average value, *k =* 1:*N.*

## 6. Results of the Three Models

The objective of the computational analysis is to build three neural network models (ANN, PSO-ANN, ASAPSO-ANN) to predict the compressive strength of rubber concrete at 28 days using a database containing 307 sample data. The model constructed and the results of the calculations are described in detail in the following subsections. Since each calculation of the artificial neural network is an approximate solution, the results for each model are averaged to avoid chance in the results. The best results from each model are selected for graphical analysis.

### 6.1. ANN Model

**Figure 6:**
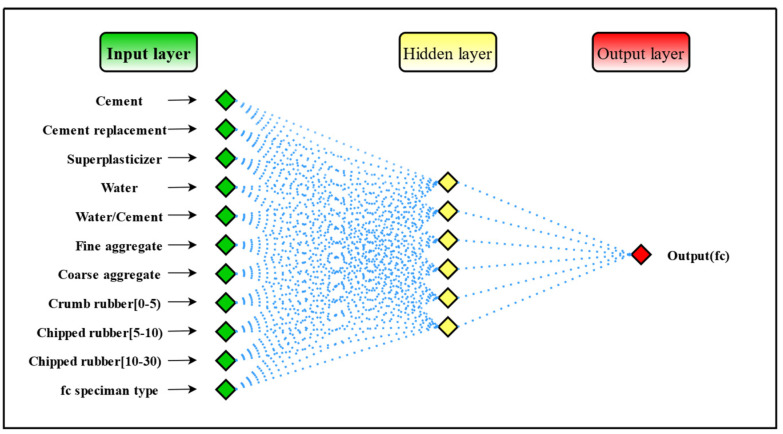
ANN structure used in this study.

A three-layer feedforward neural network model was built, and the number of neurons in the hidden layer was obtained between 4 and 13 by Equation (7), and then the trial-and-error method was used to obtain the optimal number of neurons as 6. Thus, the neural network structure was 11–6–1 (Figure 6). The parameters of the neural network model in this study are shown in Table 2.

The ANN model performance evaluation results are presented in Table 4. The average value of the training set $R^{2}$ of the ordinary ANN model was 0.8990, and the average value of the testing set $R^{2}$ was 0.8385. From these data, the $R^{2}$ for both the training and testing sets of the standard ANN model were relatively good (published literature suggests that models were high-precision when *R* > 0.8 and RMSE, MAE are low [75]). The mean value of the RMSE for the training set of the ordinary ANN model was 5.0237, and the testing set was 4.9673. The mean value of the MAE was 3.7363 for the training set and 4.2117 for the testing set. From these data, it could be seen that the common ANN model has a high error, but the RMSE and MAE values are closer. It could be concluded that the ordinary ANN model has some generalization ability and could predict the unknown data to some extent, but the prediction accuracy of the model was not high.

### 6.2. PSO-ANN Model

Weights and biases have a significant impact on the prediction results of the ordinary ANN model. Therefore, PSO algorithms were needed to find the optimal weight and biases to achieve the prediction of the target. For the PSO-ANN model, in addition to the usual parameters, the population size, number of iterations, social learning factor, individual learning factor, particle position constraint, particle velocity constraint, and inertia factor also needed to be set. The number of neurons in the hidden layer of the PSO-ANN model is also set to six. The parameter settings for the PSO-ANN in this study are shown in Table 3.

The performance evaluation results of the PSO-ANN model are shown in Table 4. The mean value of the $R^{2}$ for the training set of the PSO-ANN model was 0.9516, and the mean value of $R^{2}$ for the testing set was 0.8732. The mean value of the RMSE for the training set of the PSO-ANN model was 3.4573, and the testing set was 3.6340. The mean value of the MAE for the training set was 2.5493, and the testing set was 2.7260. From these data, the $R^{2}$ of both the training and testing sets of the PSO-ANN model improved compared to the normal ANN model ($R^{2}$ was 0.8990 for the training set and 0.8385 for the testing set of the ANN model). The RMSE for both the training and testing sets of the PSO-ANN model was reduced to varying degrees compared to the standard ANN model (RMSE was 5.0237 for the training set and 4.9673 for the testing set of the ANN model). The MAE of the training and testing sets was also lower than the ANN model (MAE was 3.7363 for the training set and 4.2117 for the testing set for the ANN model). Thus, the PSO algorithm improved the predictive ability of the ANN model.

### 6.3. ASAPSO-ANN Model

Introduction of the SA algorithm into the PSO algorithm improved the global search capability of the algorithm and the probability of jumping out of the local extremum. The inertia factor was optimized by Equation (11). For ASAPSO-ANN, the initial temperature and the temperature decay coefficient needed to be set. The initial temperature used in this study was 200 degrees, while the temperature decay coefficient was 0.95.

The results of the performance evaluation of the ASAPSO-ANN model are presented in Table 4. The mean value of $R^{2}$ for the training set of the ASAPSO-ANN model was 0.9554, and the testing set was 0.9240. The mean value of the RMSE for the training set was 3.3238, and the testing set was 2.7805. The mean value of the training MAE was 2.4016, and the mean of the testing set was 2.1088. From these data, the performance of the neural network model was further improved by adding the SA algorithm. Compared to the PSO-ANN model, the ASAPSO-ANN model has a higher $R^{2}$ on both the training and testing sets ($R^{2}$ was 0.9516 for the PSO-ANN model training set and 0.8732 for the PSO-ANN testing set), and a lower RMSE (RMSE was 3.4573 for the training set and 3.6340 for the testing set of the PSO-ANN model). The mean value of MAE decreased from 2.5493 to 2.4016 for the training set and from 2.7260 to 2.1088 for the testing. The $R^{2}$ of the testing set of the ASAPSO-ANN model was significantly higher than that of the PSO-ANN model, while other error metrics were also lower than that of the PSO-ANN model. This demonstrated that the ASAPSO algorithm further improved the predictive ability of the ANN model.

### 6.4. Weights and Biases of Neural Networks for the Three Models

This section gives the weight matrices for the three neural network models so that the neural network models can be applied. The weight matrices for the ANN model, the PSO-ANN model, and the ASAPSO-ANN model are shown in Appendix A.

## 7. Discussion

This study focused on the prediction of the compressive strength of rubber concrete using a neural network optimized by the ASAPSO algorithm. Since a single optimization algorithm has its shortcomings, it was necessary to mix the algorithms to compensate for their shortcomings between them. In the previous section, the results of the three models ANN, PSO-ANN, and ASAPSO-ANN are presented in Table 4, but it was necessary to discuss the results from a more intuitive point of view.

**Figure 7:**
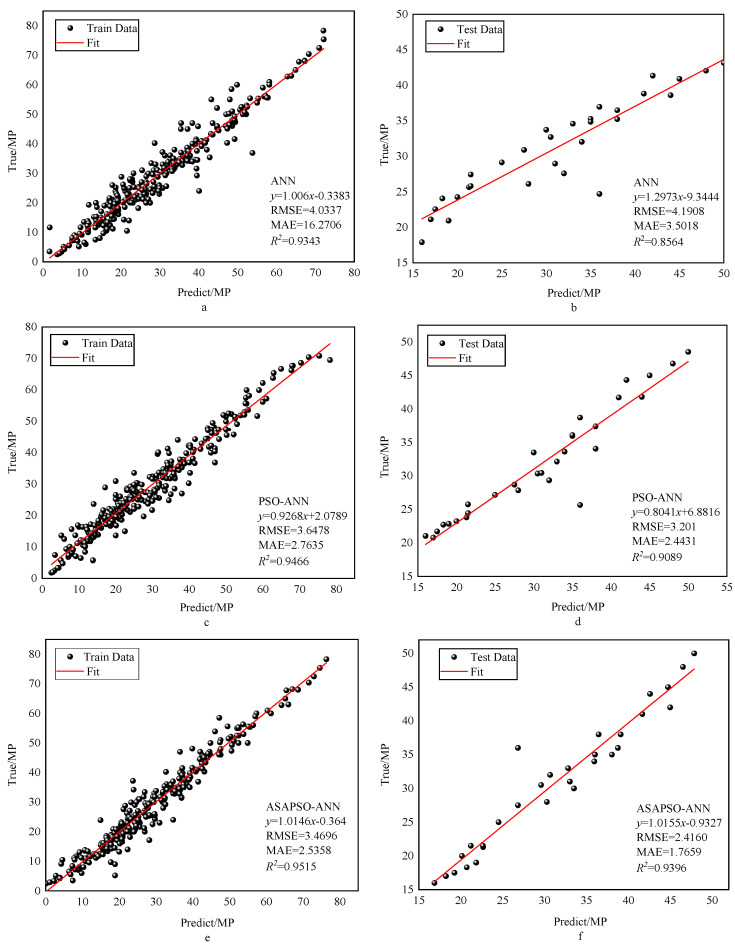
Regression analysis of the three models: (**a**,**b**) for the ANN model; (**c**,**d**) for the PSO-ANN model; (**e**,**f**) for ASAPSO-ANN model, for training and testing phases, respectively.

**Figure 8:**
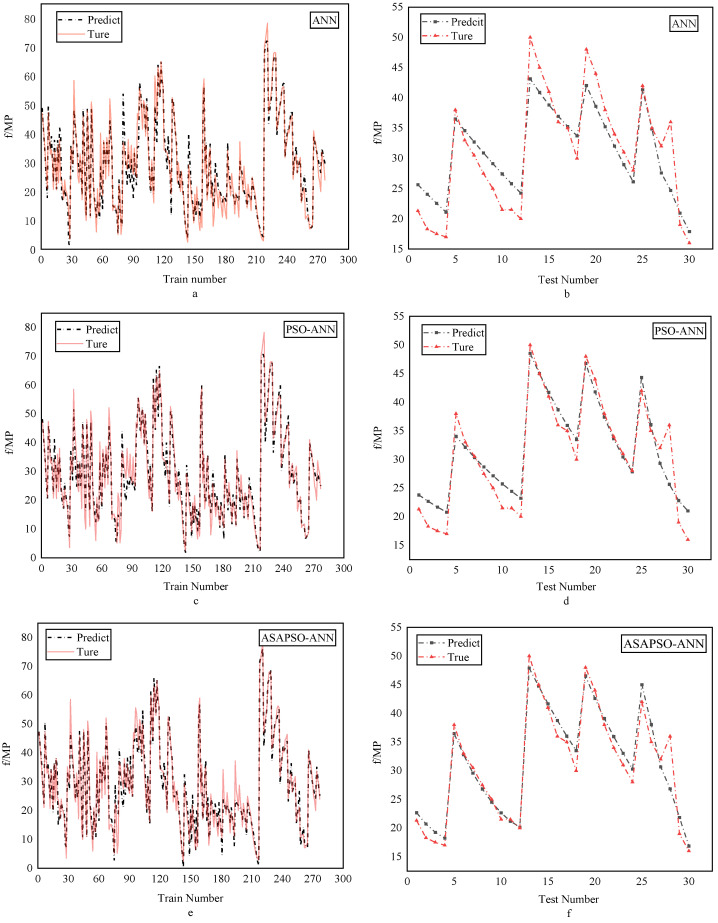
Comparison of actual and predicted values for the three models: (**a**,**b**) for ANN model; (**c**,**d**) for PSO-ANN model; (**e**,**f**) for ASAPSO-ANN model, for training and testing phases, respectively.

**Figure 9:**
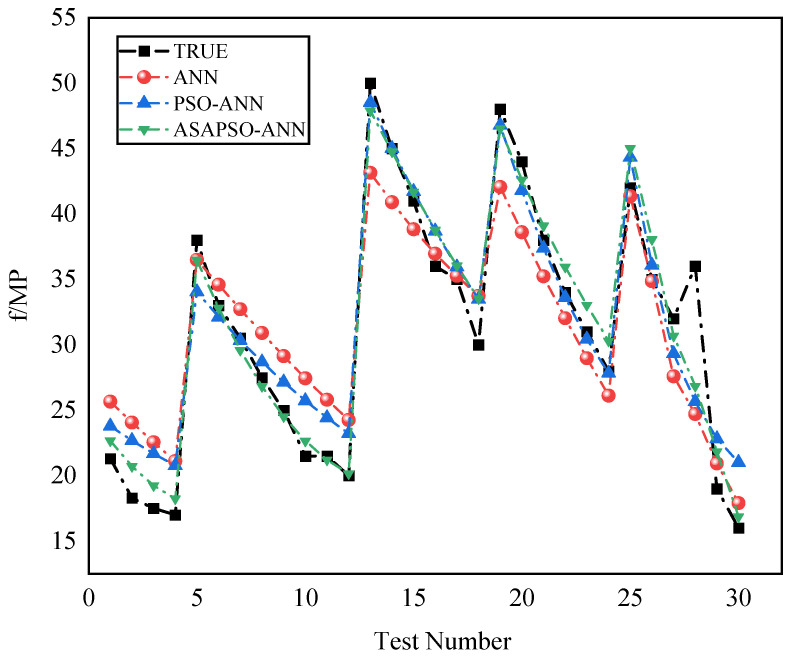
Comparison of data from three model testing sets.

**Figure 10:**
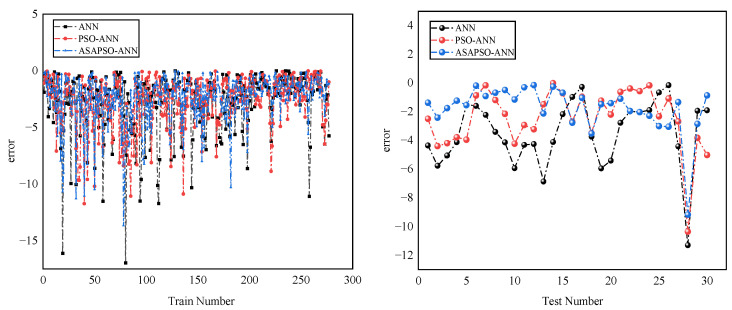
Comparison of errors in the training (**right**) and testing (**left**) sets of three models.

The regression analysis for the three models are presented in Figure 7. Panels a, c, and e denote training set results, and b, d, and f denote testing set results. The figure shows that most of the data points in the training set of the three models were distributed on both sides of the fitted line and the $R^{2}$ were good. This indicates that all three neural network models had a good fitting ability. Among these, the ASAPSO-ANN model had the highest $R^{2}$, which is the indication of a stronger fitting ability. The results from Figure 7b,d,f showed a significant increase in $R^{2}$ in the testing set compared to the training set, which increased from 0.8564 for the ANN model to 0.9396 for the ASAPSO-ANN model. Meanwhile, the ASAPSO-ANN model had the lowest error in each. Since the testing set did not participate in the training of the model, it could be shown that the ANN model with the introduction of the ASAP SO algorithm had a substantial improvement in predictive ability. This conclusion could also be drawn from Figure 8 and Figure 9. As can be seen in Figure 8f and Figure 9, the predicted value of the ASAPSO-ANN model was closer to the actual value compared to the ANN and PSO-ANN models. The error analysis for the three models is shown in Figure 10.

In summary, the introduction of the algorithm proved to be successful and effective. The PSO algorithm optimized the weights and biases of the ANN model using its optimization capabilities, resulting in improved model performance and reduced prediction errors. The introduction of the SA algorithm in the PSO algorithm successfully compensated for the shortcomings of the PSO algorithm in terms of fast convergence at the end of iteration and the tendency to fall into local extremum, so that the probability of obtaining the globally best model was greatly increased. It can also be seen that the adaptive treatment of inertia factor successfully helped the PSO algorithm to perform sufficient optimization search in the early stage and converge to the global best quickly in the later stage. This shows that the hybrid algorithm improved the prediction accuracy of the model more than the single optimization algorithm. It also shows that the hybrid algorithm could be used to study the strength of rubber concrete.

**Figure 11:**
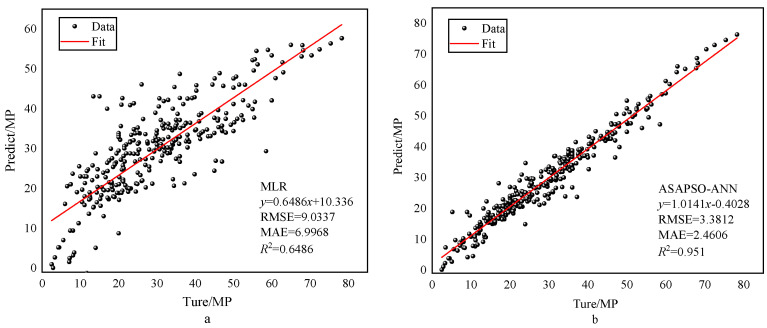
Results of regression analysis of two models: (**a**) for MRL and (**b**) for ASAPSO-ANN.

**Figure 12:**
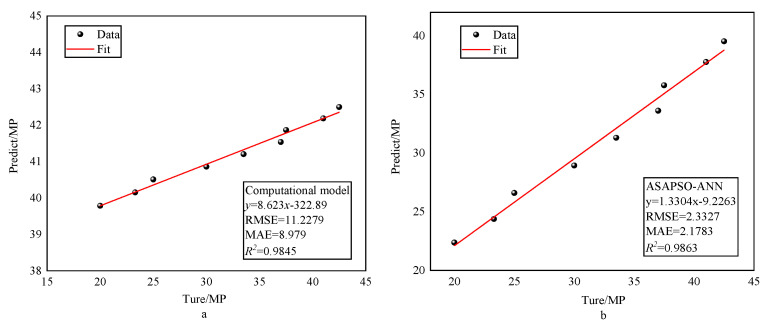
Results of regression analysis of two models: (**a**) for empirical formula and (**b**) for ASAPSO-ANN.

To further validate the reliability of the proposed model, the traditional multiple linear regression model was introduced here [17]. The multiple linear regression (MLR) model was similar to the neural network model in that both studied the effects of multiple variables; therefore, the two models could be used for comparison. The results of the regression analysis of the two models are shown in Figure 11. A comparison of the indicators is shown in Table 5. As can be seen from Figure 11 and Table 5, the ASAPSO-ANN model outperforms the MLR model. Comparison with empirical formulae was also necessary. M. Reda Taha modeled the empirical formula using polynomials [26]. Since the empirical formula only used the percentage of rubber content as an input variable, the input variable for the ASAPSO-ANN model was also changed to the percentage of rubber content. To avoid complex calculations, a randomly selected cited study [39] was used for calculations using both models. Figure 12 shows the results of the regression analysis for both models. A comparison of the indicators is shown in Table 6. From Figure 12a, it can be seen that the empirical equation model had a high $R^{2}$, but its horizontal and vertical values were very different. This indicates that there was a large error between the predicted and actual values. This is also reflected in the other indicator values in Table 6. This indicates the poor predictive ability of the empirical formula model. In contrast, the ASAPSO-ANN model represented in Figure 12b did not suffer from such problems and could predict the strength values of the concrete with accuracy. For these types of empirical formulas, the influence of multiple factors on strength was usually not taken into account, and therefore the ASAPSO-ANN model had a wider application capability than the empirical formulas. Comparison of other neural network models were also necessary. The comparison results are shown in Table 7. As can be seen from Table 6, ASAPSO-ANN performed better than the conventional ANN model when faced with large datasets. Therefore, the proposed model is feasible for strength studies of rubber concrete.

## 8. Conclusions and Future Prospect

In this study, an ASAPSO-ANN model was developed for predicting the compressive strength of rubber concrete. The data were obtained from the published literature. Both ANN and PSO-ANN artificial neural network models were developed for comparative analysis. RMS, $R^{2}$, MSE, and MAE were used to evaluate the model performance. According to the results of the testing phase, all three artificial neural networks established in this study have predictive capabilities, but the proposed ASAPSO-ANN model has the highest prediction accuracy compared to the ANN model and the PSO-ANN model ($R^{2}=0.9240$, RMS = 2.7805, MSE = 7.8028, and MAE = 2.1088). By comparing the prediction curves of the testing set of the three models, it can also be obtained that the prediction curves of the ASAPSO-ANN model are closer to the actual curves. This led to the conclusion that the SA algorithm successfully compensates for the shortcomings of the PSO algorithm, proving the validity of the proposed model.

In summary, this study successfully demonstrated that the proposed ASAPSO algorithm improved the performance and accuracy of the network. It also showed that hybrid algorithm could be used to optimize neural networks for predicting the strength of rubber concrete. This provided a new option for predicting the strength of rubber concrete using neural network models. However, observation of the results revealed that the proposed method could be further optimized in the future to obtain better results, which included making the dataset more comprehensive, considering the issue of outliers in the model, and comparing it with other machine learning models.

## Figures and Tables

**Table 1:** Statistical analysis of input and output variables.

	Max	Min	Average	Median	Standard Deviation	Skewness
Cement (kg/m^3^)	629.27	18.80	406.81	400.00	75.22	−0.26
Cement replacement (kg/m^3^)	180.00	0.00	12.19	0.00	31.84	3.38
Superplasticizer (kg/m^3^)	13.50	0.00	3.23	2.08	3.96	1.45
Water (kg/m^3^)	312.00	9.20	192.83	180.00	38.43	−0.24
Water/cement (kg/m^3^)	0.83	0.27	0.48	0.45	0.11	0.67
Fine aggregate (kg/m^3^)	1364.00	0.00	610.11	631.37	219.09	−0.63
Coarse aggregate (kg/m^3^)	1434.60	0.00	911.54	949.00	230.99	−0.44
Crumb rubber [0–5) (kg/m^3^)	1160.00	0.00	59.06	36.00	102.16	6.56
Chipped rubber [5–10) (kg/m^3^)	227.30	0.00	14.46	0.00	40.29	3.55
Chipped rubber [10–30) (kg/m^3^)	630.00	0.00	16.40	0.00	59.47	6.52
fc specimen type	3.00	0.00	0.89	1.00	0.73	0.63
fc (MP)	78.30	2.50	29.41	27.05	15.24	0.65

**Table 2:** Parameter setting of ANN model.

Parameter	Setting
Input layer node	11
Output layer node	1
Hidden layer node	6
Activation function	Tansig, purelin
Training function	trainlm
Epochs	50
Learning rate	0.01
Performance goal	1.00 × 10^−5^
Epochs between display	25
Momentum factor	0.01
Minimum performance gradient	1.00 × 10^−6^
Maximum validation failure	6

**Table 3:** Parameter settings for the PS0-ANN model.

Parameter	Setting
Popsize	15
Maxgen	500
$c_{1}$	2
$c_{2}$	2
ω	0.95
Position constraint	[−3,3]
Velocity constraint	[−3,3]

**Table 4:** Results of the performance evaluation of the three models.

		ANN	PSO-ANN	ASAPSO-ANN
$R^{2}$	Train	0.8990	0.9516	0.9554
	Test	0.8385	0.8732	0.9240
MSE	Train	26.8847	12.0370	11.0969
	Test	25.1023	13.3453	7.4011
RMSE	Train	5.0237	3.4573	3.3238
	Test	4.9673	3.6340	2.7805
MAE	Train	3.7363	2.5493	2.4016
	Test	4.2117	2.7260	2.1088

**Table 5:** Metrics for MLR and ASAPSO-ANN models.

	$R^{2}$	MSE	RMSE	MAE
ASAPSO-ANN	0.951	11.4323	3.3812	2.4606
MLR	0.6486	81.6069	9.0337	6.9968

**Table 6:** Metrics for empirical formula and ASAPSO-ANN models.

	*R* ^2^	MSE	RMSE	MAE
ASAPSO-ANN	0.9863	5.4414	2.3327	2.1783
M. Reda Taha [26]	0.9845	126.0657	11.2279	8.979

**Table 7:** Comparison with models in the literature.

	ML Algorithm	Structure	Dataset	Performance
This study	ASAPSO algorithm with ANN	11–6–1	307	R = 0.9774(train)
				R = 0.9612(test)
				R = 0.9752(all)
Khoja [9]	ANN with Levenberg–Marquardt algorithm	5–10–1	287	R = 0.954(all)
Abdollahzadeh [17]	ANN multi-layered perceptron (BP)	3–1–1	20	R = 0.9885(train)
				R = 0.9824test)

## Data Availability

The data used in the article can be obtained from the author here.

## Appendix A

ANN:

$$w1=\left[\begin{array}{ccccccccccc} 0.130 & -0.636 & -0.410 & 0.336 & -0.860 & -0.00 & 0.439 & -0.130 & -0.700 & -0.096 & -0.751\;\\ -0.102 & 0.470 & 0.171 & 0.605 & 0.757 & -0.362 & 0.373 & 0.725 & 0.673 & -0.466\; & -0.220\;\\ 0.421 & 0.603 & -0.459 & -0.307 & -0.613 & -0.540 & 0.411 & 0.298 & -0.704 & 0.571 & 0.350\;\\ -0.231 & -0.037\; & -0.460 & 0.668 & -0.428 & 0.637 & -0.545 & -0.640 & -0.581 & 0.588 & -0.155\;\\ -0.835 & -0.370 & \;0.098 & \;0.018\; & 0.328 & 0.153 & -0.630 & -0.874 & -0.022 & 0.660 & -0.378\;\\ -0.042 & 0.474 & -0.618 & 0.360 & 0.467\; & -0.439 & 0.198 & -0.601 & -0.621 & -0.612 & -0.636\; \end{array}\right]\;$$

w2=[−0.235−0.249−0.768−0.799 0.014−0.885]

$$b1={\left[\begin{array}{cccccc} -1.648 & 0.989 & -0.330 & -0.330 & -0.986 & -1.648 \end{array}\right]}^{T}$$

$$b2={\left[-0.227\right]}^{T}$$

PSO-ANN:

$$w3=\left[\begin{array}{ccccccccccc} -2.428 & -0.857 & -0.303 & 2.257 & -1.039 & 0.364 & -0.159 & \;5.346 & 0.870 & 2.751 & -0.311\;\\ 3.711 & 1.442 & 4.023 & 2.092 & 0.645 & 3.596 & -1.585 & -0.736 & 0.133 & 2.603 & 2.975\;\\ 2.762 & 1.015 & -0.196 & -1.843 & 0.933 & -1.459 & -0.184 & -4.372 & -5.070 & -2.021 & 0.706\;\\ 0.970 & 1.115 & 1.016 & 5.295 & -0.281 & 3.921 & 7.953 & -2.226 & -0.617 & 1.082 & 9.331\;\\ 0.871 & 3.674 & 3.767 & \;3.616\; & -1.162 & -2.082 & 4.125 & -3.288 & -2.313 & -3.038 & 0.561\;\\ 7.484 & 0.425 & 6.608 & 13.598 & -3.780\; & \;5.058 & -3.747 & 1.662 & -2.279 & -2.991 & -0.761\; \end{array}\right]$$

w4=[−1.380−2.93−1.557 0.209−0.040 0.064]

$$b3={\left[\begin{array}{cccccc} 8.195 & -1.706 & -12.078 & 6.403 & 2.066 & -2.192 \end{array}\right]}^{T}$$

$$b4={\left[-3.921\right]}^{T}$$

ASAPSO-ANN:

$$w5=\left[\begin{array}{ccccccccccc} -5.804 & -2.772 & -1.222 & \;0.163 & -0.356 & -2.316 & 1.700 & \;3.703 & 0.884 & 3.381 & 3.198\;\\ -4.767 & -0.563 & -0.436 & -0.586 & 2.726 & \;2.958 & 6.314 & -6.992 & 3.005 & 6.417 & -0.152\\ -2.509 & 1.148 & -1.844 & -3.255 & 1.088 & \;1.858 & -0.074 & -1.452 & 1.547 & 1.895 & 2.816\\ 1.370 & 1.195\; & -2.008 & -4.159 & 1.528 & -4.568 & -2.848 & -2.623 & -1.290 & 1.446 & -0.018\\ 0.445 & 0.224 & \;0.339 & -0.882 & 0.135 & \;0.082 & 0.316 & -3.514 & -0.743 & -2.287 & 0.008\\ 3.672 & 0.538 & -1.742 & -2.114 & 0.364 & -4.714 & 2.701 & 0.646 & -0.439 & 2.871 & 6.128\; \end{array}\right].\;$$

w6=[−0.250 0.290−0.091−0.283 1.944 0.288]

$$b5={\left[\begin{array}{cccccc} 7.154 & -1.294 & -3.143 & -2.614 & -7.033 & 4.742 \end{array}\right]}^{T}$$

$$b6={\left[1.240\right]}^{T}$$
